# Accuracy of prostate cancer screening recommendations for high‐risk populations on YouTube and TikTok

**DOI:** 10.1002/bco2.200

**Published:** 2022-11-08

**Authors:** Max Abramson, Nathan Feiertag, Darius Javidi, Mustufa Babar, Stacy Loeb, Kara Watts

**Affiliations:** ^1^ Albert Einstein College of Medicine Bronx New York USA; ^2^ Departments of Urology and Population Health New York University Langone Health and Manhattan Veterans Affairs Medical Center New York New York USA; ^3^ Department of Urology, Montefiore Medical Center Albert Einstein College of Medicine Bronx New York USA

**Keywords:** high‐risk populations, misinformation, patient education, prostate cancer screening, racial disparities, social media, TikTok, YouTube

## Abstract

**Objectives:**

This study aimed to evaluate content quality and racial/ethnic representation, particularly of high‐risk cohorts, of prostate cancer screening videos on YouTube (YT) and TikTok (TK).

**Materials and Methods:**

The top 50 videos populated for the search term ‘prostate cancer screening’ on YT and TK that met inclusion criteria were retrieved in a cache‐cleared browser. Three reviewers analysed all videos using validated criteria for the quality of consumer health information (DISCERN and Patient Education Materials Assessment Tool [PEMAT]). High quality was defined as follows: DISCERN ≥ 4, PEMAT understandability ≥75% and PEMAT actionability ≥75%. A 5‐point Likert scale was used to demonstrate the level of misinformation compared to American Urological Association and National Comprehensive Cancer Network guidelines. Perceived race and ethnicity of people in the videos were assessed by consensus approach.

**Results:**

TK videos were shorter (median 3.7 vs. 0.5 min, *p* < 0.001) and had more views per month (5437.5 vs. 19.3, *p* = 0.03) than YT videos. Perceived Black and Hispanic representation was present in 10% and 6% of YT videos and 20% and 12% of TK videos, respectively. High‐risk racial/ethnic groups were explicitly discussed in 46% of YT videos and 8% of TK videos. A total of 98% of YT videos and 100% of TK videos had low‐ to moderate‐quality consumer health information, and 88% of YT videos and 100% of TK videos had moderate to high levels of misinformation based on screening guidelines.

**Conclusions:**

YT and TK videos about prostate cancer screening are widely viewed but do not provide quality consumer health information. Black and Hispanic men remain under‐represented on both platforms, and high‐risk racial groups were not discussed in most videos despite the importance for screening criteria. The low understandability and actionability, significant misinformation and lack of diversity in online videos support the need for higher quality videos with adequate attention to high‐risk ethnic cohorts.

## 1 INTRODUCTION

As we continue to progress into a more technologically dependent and savvy society, individuals and healthcare providers are utilizing the internet and social media for healthcare information and medical advice. In fact, the percentage of American adults using social media increased substantially from 5% in 2005 to 72% in 2021.^1^ Given that 8 in 10 American adults use the internet to search for health information,^2^ the impact of social media on medical decision‐making is likely considerable.^3^

Black men have a higher incidence and mortality from prostate cancer than White men,^4^, ^5^ and Hispanic men have an increased risk of advanced stage disease.^6^ Guidelines pertaining to prostate cancer screening and treatment largely support the concept of shared decision‐making,^7^, ^8^ which places an expectation on physicians to enable patients to play a more active role in their medical care.^9^ Numerous studies have demonstrated inequalities and disparities among racial and ethnic groups in terms of prostate cancer screening,^10^ a number of which have shown Black and Hispanic men to be disproportionately and negatively impacted compared to White men. Although the factors contributing to this disparity are likely multilevel, one component may be attributable to higher levels of physician distrust among Black and Hispanic men.^11^, ^12^ Indeed, prior studies suggest that Black and Hispanic men may be more likely to seek out and trust health information online.^9^, ^13^

The ability of online content to conform to its audiences' identity is crucial for positive evaluations of health information.^14^ Therefore, under‐representation of Black and Hispanic men in online content may limit the accessibility of and viewer identification with the information. Additionally, few Black men perceive themselves to be at a higher risk of developing prostate cancer^15^ than the general population, despite the statistics to the contrary.^4^, ^5^ Therefore, accurate and accessible prostate cancer screening information tailored to racially and ethnically diverse populations could play a role in encouraging these men to seek screening at an earlier and more appropriate stage.

YouTube (YT; subsidiary of Google) and TikTok (TK; ByteDance Ltd.) currently represent the most popular video‐based social media platforms.^1^ YT is the second largest search engine, amassing 30 million daily users worldwide^16^ and utilized by 81% of adults in the United States, with the highest use among Hispanic (85%) and Black adults (84%).^1^ Similarly, TK accrues more than one billion active users each month,^17^ including 31% of Hispanic and 30% of Black adults in the United States.^1^ A recent study has shown that patients who rely on the internet as their primary source of health information for prostate cancer are significantly more likely to report decisional regret and a worse‐than‐expected overall treatment experience.^18^ Furthermore, many recent studies have found social media and online content about various urologic conditions to be low quality and inaccurate.^19^, ^20^, ^21^, ^22^, ^23^, ^24^, ^25^, ^26^ Given the expansive reach of both YT and TK, it is important to understand the scope of health information that is being made publicly available via these platforms.

Despite the increasing role that social media plays in disseminating health information, few studies have evaluated the impact of social media on prostate cancer screening, particularly among high‐risk cohorts.^27^, ^28^, ^29^, ^30^, ^31^ Therefore, we aimed to analyse and compare YT and TK videos focused on prostate cancer screening in order to determine whether they represent racial and ethnic diversity, accurately reflect guidelines pertaining to high‐risk cohorts and meet validated quality criteria for consumer health information.

## 2 MATERIALS AND METHODS

### 2.1 Search strategy

On 12 August 2021, a cookie‐free, cache‐cleared, incognito Safari browser was used to obtain the top 50 videos for the search term ‘prostate cancer screening’ on both YT and TK. The default search settings of both platforms were used to mimic the most likely use case of a standard user in the general population. Videos in languages other than English or with no accompanying audio, duplicated content and videos unrelated to prostate cancer were excluded (Figure S1). Videos longer than 12 min were also excluded as viewer engagement significantly reduces in lengthier videos.^32^

### 2.2 Video parameters and evaluations

Data collected for each video included number of views, comments, likes, dislikes (YT only) and shares (TK only), as well as video length and date of publication. The number of months since publication and number of views per month were calculated.

Three reviewers (M.A., N.F. and D.J.) independently analysed each video to determine if it included the following content: recommendations for high‐risk racial/ethnic cohorts, recommendations for family history of prostate cancer, prostate‐specific antigen (PSA) testing, blood tests other than PSA, genomic testing, digital rectal examination, age‐specific screening recommendations, magnetic resonance imaging of the prostate for screening and targeted/fusion prostate biopsy.

Furthermore, a consensus approach was used to determine the perceived race and ethnicity of the non‐animated humans in each video.^33^ Racial categories included were Black, White, Asian, mixed race, other and unable to discern, whereas ethnic categories included Hispanic/Latino, non‐Hispanic/Latino and unable to discern. Given the inherently subjective nature of perceived race and ethnicity, we used an unequal number of reviewers to determine the perceived race and ethnicity of the non‐animated humans via majority vote.

The same three reviewers analysed the quality of consumer health information using two validated instruments, DISCERN and audiovisual version of Patient Education Materials Assessment Tool (PEMAT). DISCERN^34^ is a standardized set of criteria for evaluating quality of health information, and PEMAT^35^ is a systematic method to analyse understandability and actionability of health information. DISCERN is scored on a 5‐point scale, whereas PEMAT is a set of binary questions that results in a final percentage as a score. The total DISCERN score, PEMAT understandability percentage and PEMAT actionability percentage for each reviewer were averaged to determine a final mean score for each video. A higher score (DISCERN) and percentage (PEMAT) corresponds to higher quality overall. Videos were determined to be ‘high quality’ if they received an average score among all reviewers of DISCERN ≥ 4, PEMAT understandability ≥75% and PEMAT actionability ≥75%. To assess accuracy of information, a 5‐point Likert scale adapted from previously published studies^36^ (1 = *strongly disagree*; 5 = *strongly agree*) was used to respond to the statement, ‘this video does not contain misinformation’, when compared to the most recent guidelines set forth by the American Urological Association (AUA)^7^ and the National Comprehensive Cancer Network (NCCN).^8^ Videos were determined to contain accurate information if they received an average score among all reviewers of Likert ≥4 relative to at least one set of guidelines.

### 2.3 Statistical analysis

SPSS Version 27 (Chicago, IL, USA) was utilized for data analysis. Descriptive statistics were performed using Mood's median test to characterize the YT and TK cohorts by median views, likes and comments. Chi‐squared tests were performed to compare the frequencies of inclusion of high‐quality video content and information, such as discussion of screening guidelines for high‐risk groups, additional screening options and high‐quality DISCERN and PEMAT scores. This methodology was also applied when performing the analysis between racial cohorts within the YT group. A *p* value <0.05 was considered statistically significant, and all tests were two‐sided.

## 3 RESULTS

**FIGURE 1:**
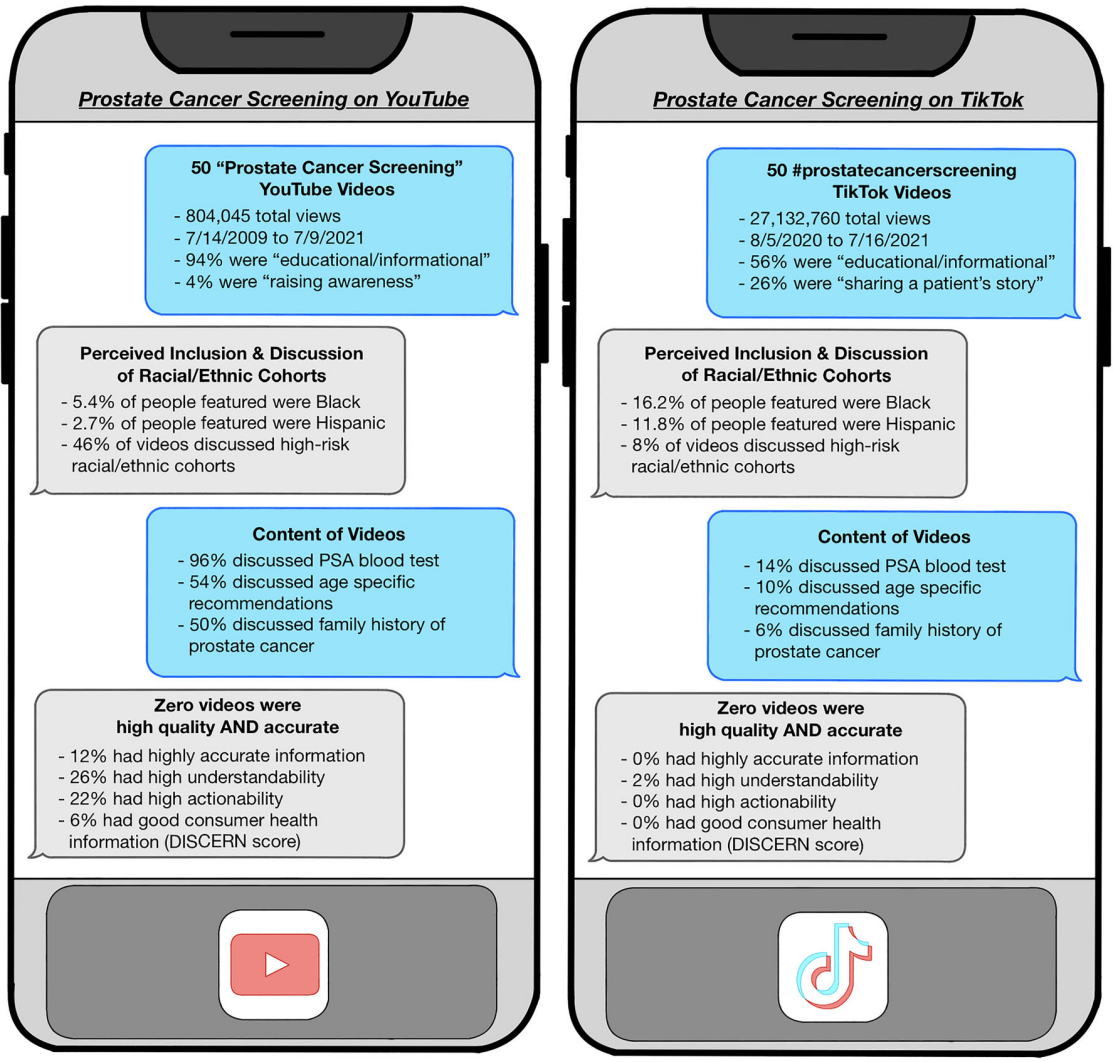
Summary of prostate cancer screening content on YouTube and TikTok. PSA, prostate‐specific antigen

A total of 62 YT and 75 TK videos were reviewed in order to obtain 50 videos on each platform that met inclusion criteria (Figure S1). Figure 1 highlights the key characteristics of the videos on each platform. YT videos received a median number of 909 views (range: 12–221 003), 7 likes (range: 0–1000) and 0 comments (range: 0–148), whereas TK videos received a median number of 23 150 views (range: 640–13 900 000, *p* = 0.07), 576 likes (range: 3–2 300 000, *p* = 0.25) and 32 comments (range: 0–13 500, *p* = 0.08) (Table 1). Nearly half of the YT videos (46%) were published more than 5 years ago, whereas all the TK videos were published within 1 year prior to data extraction. The breakdown of YT videos by genre classified 94% as ‘educational/informational’, 4% as ‘raising awareness’, 2% as ‘sharing a patient's story’ and 0% as ‘comedy’. Of the TK videos, 56% were classified as ‘educational/informational’, 26% as ‘sharing a patient's story’, 10% as ‘comedy’ and 8% as ‘raising awareness’.

**TABLE 1:** Video characteristics

Parameters	YouTube	TikTok	*p* value
Median video length (mm:ss), *n* (range)	03:42 (00:30–12:06)	00:29 (00:10–00:49)	<0.001
Median views, *n* (range)	909 (12–221 003)	23 150 (640–13 900 000)	0.07
Median views per month, *n* (range)	19.30 (0.52–20369.50)	5437.50 (177.38–3 475 000)	0.03
Median likes, *n* (range)	7 (0–1000)	576 (3–2 300 000)	0.3
Median comments, *n* (range)	0 (0–148)	32 (0–13 500)	0.08
Year published, *n* (%)			<0.001
Before 2015	20 (40)	0 (0)	
2015 and after	30 (60)	50 (100)	
Video subject representation, *n* (%)			
White representation	44 (98)	24 (52)	<0.01
Black representation	5 (10)	10 (20)	0.16
Hispanic representation	3 (6)	6 (12)	0.3
Number of videos that reference guideline, *n* (%)			<0.001
USPSTF	13 (26)	0 (0)	
AUA	5 (10)	0 (0)	
NCCN	6 (12)	0 (0)	
Other	4 (8)	0 (0)	
None	30 (60)	50 (100)	
Genre of the video, *n* (%)			<0.001
Educational/informational	47 (94)	28 (56)	
Raising awareness	2 (4)	4 (8)	
Sharing a patient's story	1 (2)	13 (26)	
Comedy	0 (0)	5 (10)	

Abbreviations: AUA, American Urological Association; NCCN, National Comprehensive Cancer Network; USPSTF, United States Preventive Services Task Force.

### 3.1 Inclusion and discussion of high‐risk racial/ethnic cohorts

Out of a total 112 people featured in the YT videos, 5.4% were perceived as Black and 2.7% were perceived as Hispanic. In contrast, TK featured 68 people, of which 16.2% were perceived as Black and 11.8% were perceived as Hispanic. Among the 50 videos from each platform, 10% of YT videos and 8% of TK videos did not include any people. YT videos were more likely to have White speakers than TK videos (YT: 98%, TK: 52%, *p* < 0.001). There was no significant difference between YT and TK videos for inclusion of Black or Hispanic speakers (YT: 11%, TK: 22%, *p* = 0.17; and YT: 7%, TK: 13%, *p* = 0.31, respectively). YT videos were more likely to discuss racial and ethnic groups, including Black men, at high risk for prostate cancer per AUA and NCCN guidelines (YT: 46%, TK: 8%, *p* < 0.001) (Table 2).

**TABLE 2:** Video content, quality and accuracy

Parameters	YouTube	TikTok	*p* value
Discussion of high‐risk groups, *n* (%)			
High‐risk race/ethnicity	23 (46)	4 (8.0)	<0.001
Age‐specific recommendations	27 (54)	5 (10)	<0.001
Family history recommendations	25 (50)	3 (6)	<0.001
Discussion of additional screening tools, *n* (%)			
Digital rectal exam	17 (34)	7 (14)	0.02
PSA testing	48 (96)	7 (14)	<0.001
MRI screening	6 (12)	0 (0)	0.01
Prostate biopsy	5 (10)	0 (0)	0.02
Genetic testing	5 (10)	0 (0)	0.02
Video quality, *n* (%)			
DISCERN risks score ≥4	11 (22)	0 (0)	<0.001
DISCERN benefits score ≥4	10 (20)	0 (0)	<0.001
DISCERN shared decision‐making score ≥4	14 (28)	0 (0)	<0.001
DISCERN high‐quality determination score ≥4	3 (6)	0 (0)	0.08
PEMAT understandability score ≥75%	13 (26)	1 (2)	<0.001
PEMAT actionability score ≥75%	11 (22)	0 (0)	<0.001
Accuracy of content			
Mean AUA Likert, score ± SD	2.293 ± 0.913	1.113 ± 0.354	<0.001
Mean NCCN Likert, score ± SD	2.140 ± 0.873	1.107 ± 0.333	<0.001
Mean Likert score ≥4, *n* (%)	6 (12)	0 (0)	0.01

Abbreviations: AUA, American Urological Association; NCCN, National Comprehensive Cancer Network; PEMAT, Patient Education Materials Assessment Tool; PSA, prostate‐specific antigen.

### 3.2 Content

Outside of discussing PSA testing in 55% of videos across both platforms, other specific components of prostate cancer screening guidelines were discussed in fewer than one third of videos on both platforms. YT videos were more likely to include specific recommendations for patients with a family history of prostate cancer (YT: 50%, TK: 6%, *p* < 0.001), as well as age‐specific recommendations (YT: 54%, TK: 10%, *p* < 0.001). YT videos were more likely to discuss additional screening tools to evaluate prostate cancer risk, such as digital rectal examination (YT: 34%, TK: 14%, *p* = 0.019), PSA testing (YT: 96%, TK: 14%, *p* < 0.001), magnetic resonance imaging screening (YT: 12%, TK: 0%, *p* = 0.012), prostate biopsy (YT: 10%, TK: 0%, *p* = 0.022) and genomic testing (YT: 10%, TK: 0%, *p* = 0.022).

### 3.3 Quality and misinformation

Overall, there were no videos that contained both high‐quality and accurate information. Furthermore, few videos across both platforms had good support for shared decision‐making (14%), and even fewer were deemed either sufficiently accurate (6%) or high quality (1%). YT videos were significantly more likely to receive high‐quality scores for individual components of the DISCERN criteria, including discussion of the risks (YT: 22%, TK: 0%, *p* < 0.001) and benefits (YT: 20%, TK: 0%, *p* < 0.001) of prostate cancer screening, as well as discussion of the importance of shared decision‐making (YT: 28%, TK: 0%, *p* < 0.001). Similarly, YT videos were significantly more likely to contain high‐quality information with regard to PEMAT understandability score (YT: 26%, TK: 2%, *p* < 0.001) and actionability score (YT: 22%, TK: 0%, *p* < 0.001). There were no significant differences in the number of videos that received high‐quality determination for overall DISCERN scores (YT: 6%, TK: 0%, *p* = 0.079) and that were classified as ‘high‐quality’ overall (YT: 2%, TK: 0%, *p* = 0.3) between the platforms. However, YT videos were significantly more likely to contain accurate information relative to screening guidelines (YT: 12%, TK: 0%, *p* = 0.012). No TK videos specifically cited a specific guideline, whereas 16 (32%) of YT videos cited at least one guideline (*p* < 0.001).

The frequencies of high‐quality and accurate videos were compared between YT videos with people perceived as Black and/or Hispanic (*n* = 7) versus without (*n* = 43). There were no significant differences in the frequency of videos that received high‐quality overall DISCERN scores (W/: 0%, W/out: 7%, *p* = 0.5), PEMAT understandability scores (W/: 28.6%, W/out: 25.6%, *p* = 0.9) and PEMAT actionability scores (W/: 14.3%, W/out: 23.3%, *p* = 0.6). Similarly, there were no significant differences in the frequency of videos containing accurate information according to AUA and NCCN guidelines (W/: 0%, W/out: 7%, *p* = 0.3). A similar analysis was not completed for the TK videos as there was only one TK video with a sufficiently high PEMAT understandability score and no TK videos with sufficiently high PEMAT actionability, DISCERN or Likert scores to be included.

## 4 DISCUSSION

Our study is the first to evaluate prostate cancer screening content on TK and compare it to the content on YT. We demonstrated that the overall quality of prostate cancer screening videos is higher on YT than on TK; however, videos on both platforms lacked representation of racial and ethnic diversity, particularly as it relates to high‐risk cohorts for prostate cancer. Furthermore, despite the fact that TK videos were much more widely viewed than YT videos, YT videos were more likely to contain accurate screening information and to receive high‐quality individual DISCERN scores, such as those for discussion of screening risks, benefits and the importance of shared decision‐making. Nevertheless, neither platform provided high‐quality consumer health information.

Several studies have evaluated prostate cancer screening content on YT, all of which found the information to be biased, of poor quality and potentially misleading.^27^, ^28^, ^29^, ^31^ Interestingly, Shungu et al. evaluated the quality of information regarding prostate cancer screening on YT for Black men. Similar to our study, Shungu et al. found that less than half of videos addressed racial disparities in prostate cancer but no difference in quality of the content based on perceived race of the presenter.^28^ Although there have been no studies that have evaluated prostate cancer screening content on TK, there has been one study that has evaluated general prostate cancer content on TK.^36^ In this study, Xu et al. found that most TK videos focused on raising awareness or paying tribute to specific individuals with prostate cancer, and of the few videos with educational content, about half of them contained significant misinformation.^36^ Our study builds on the work from these previous studies by directly comparing prostate cancer screening content on these two popular video‐sharing platforms and with a focus on the inclusion of specific screening recommendations for Black and Hispanic cohorts.

Even though YT was more likely to contain accurate information than TK, most YT videos and all TK videos had moderate to significant levels of misinformation when compared to AUA and NCCN prostate cancer screening guidelines.^7^, ^8^ There may be a bias when comparing content on the two platforms because YT videos have no length restrictions, whereas TK videos were limited to 3 min at the time of data collection. However, the utility of our analysis is not limited to comparing the length of the videos on the two platforms. Instead, we aimed to objectively quantify the quality and accuracy of prostate cancer screening videos on each platform. Two crucial components inherent in this analysis of accuracy are which guidelines the creators of the video chose to use and when the video was published. The high levels of misinformation seen throughout the YT videos may partially be explained by the fact that almost half of the videos were published more than 5 years ago, and prostate cancer guidelines change over time. Once a YT video is published, it is rare for the publisher to go back and retract the video, as evidenced by the fact that so many of the YT videos were published more than 5 years ago; thus, the video will continue to be recommended to viewers long after the information has become outdated. However, the same logic cannot currently be applied to TK as it is a newer platform. Nevertheless, this challenge will continue to be inherent in social media and be a source of misinformation for viewers in the future.

Our study focused exclusively on video‐sharing platforms as a source of information for patients; however, YT and TK exist within the wider context of the internet. In a recent study, Gunasegaram et al. reviewed 5400 webpages to evaluate the quality of online urology information.^37^ They found that online information frequently lacks validation and is of indeterminate credibility. Similarly, Loeb et al. compared the quality of prostate cancer information on 150 YT videos with that on 150 websites and found that most content did not meet quality criteria for health information.^33^ More specifically, two YT videos and zero websites were the appropriate reading level for consumers *and* met quality criteria guidelines. These studies help contextualize that online content, in all its forms, comes with limitations due to the lack of control and oversight of what is posted. These challenges are not exclusive to YT and TK; however, they are inherent within YT and TK, which further emphasizes the importance of quantifying the quality and accuracy of the health information readily available to patients. One area where video‐sharing platforms and websites differ is that websites are able to publicize their credibility by earning certification through Health on the Net Foundation Code of Conduct (HONcode).^38^ We believe that a similar certification for content creators on video‐sharing platforms would be helpful for identifying higher quality health information.

Overall, we found that YT videos with diverse racial and/or ethnic representation were not significantly different from those without representation in terms of the quality of consumer health information or level of misinformation. A similar analysis for TK videos was not completed due to only one TK video having a sufficiently high understandability score and no TK videos with sufficiently high actionability, DISCERN or Likert scores. Because YT videos are lacking in both their quality *and* their diversity, increasing representation alone would be unlikely to result in high‐quality consumer health information. Compared to the 2020 United States Census and US prostate cancer survivors, Black and Hispanic representation was lower on both YT and TK.^39^, ^40^ Lack of diversity in these videos is concerning because Black and Hispanic men may be less likely to report positive evaluations.^14^, ^41^ Importantly, there are currently ongoing research studies to evaluate the impact that under‐representation has on decision‐making about prostate cancer.

This study has a few limitations to acknowledge. We only reviewed videos in the English language, and future studies are warranted to examine Spanish‐language content given the importance of screening in the Hispanic population. Furthermore, we limited the length of videos to less than 12 min, which may have led to a selection bias. However, other studies that analysed prostate cancer information on YT and did not limit video length also found low‐quality information and concern about content accuracy.^27^, ^31^ Additionally, AUA and NCCN guidelines for prostate cancer screening were used as our reference guidelines; however, many other groups (e.g., United States Preventive Services Task Force and European Association of Urology) also have prostate cancer screening guidelines. That said, shared decision‐making has become a common feature across current guidelines, and AUA and NCCN were chosen as commonly utilized by practising urologists in the United States. Despite our efforts to maintain objectivity with a consensus approach among reviewers, examining race/ethnicity is inherently subjective.^33^ Furthermore, data analysis on stratified sub‐groups of videos was limited by statistical power. This could potentially be mitigated by having a larger sample size of videos; however, there is an issue of diminishing utility, as it becomes increasingly unlikely that a layperson would select a video recommended so far down by the YT or TK algorithm.^42^ Additionally, our study is a cross‐sectional analysis, and therefore, the top results that appear when prostate cancer screening is searched may change due to the dynamic nature of the content on these platforms. In spite of these limitations, our study represents an accurate representation of the current state of consumer information on YT and TK regarding prostate cancer screening.

Given the emphasis placed on early screening of high‐risk cohorts by the AUA and NCCN, the misinformation and poor quality of YT and TK videos may be particularly harmful to Black and Hispanic men. Therefore, we advocate for a concerted effort from healthcare professionals and organizations, including the AUA and NCCN, to publish diverse, high‐quality and accurate videos about prostate cancer screening on YT and TK. Digital marketing experts may also need to be consulted to help these higher quality content quickly gain attraction and become more popular than the outdated content. Furthermore, there should be a push to delete videos that give recommendations from outdated guidelines. Finally, with the knowledge that patients will likely seek out information online, and particularly on YT or TK, physicians should familiarize themselves with high‐quality content creators to recommend appropriate resources to their patients. These changes would help patients become better informed about their health, so they can actively engage in shared decision‐making with their physicians regarding prostate cancer screening.

## 5 CONCLUSION

In conclusion, none of the 100 videos analysed on YT or TK offered understandable, actionable, accurate *and* high‐quality consumer health information about prostate cancer screening. Furthermore, Black and Hispanic adults were under‐represented on both platforms. YT videos were more likely to discuss the importance of screening in high‐risk racial/ethnic cohorts, as well as to be understandable, actionable *or* accurate; however, TK video had a larger viewership. Ultimately, this results in the wide dissemination of subpar prostate cancer screening information that may contribute to disparities in prostate cancer screening. Therefore, given the widespread use of social media by patients for healthcare information, we recommend a collaborative effort from the medical community to create high‐quality content regarding prostate cancer screening that also represents those patients who are most at risk.

## DISCLOSURE OF INTEREST

None.

## AUTHOR CONTRIBUTIONS

All authors prepared the manuscript and figures and approved the submitted manuscript.

## Supporting information


**Figure S1.** Flowchart of video inclusion and breakdown of general findings on YouTube and TikTok
