# Supplementary figures and images for: Accuracy of prostate cancer screening recommendations for high‐risk populations on YouTube and TikTok
Source: BJUI Compass. 2022 Nov 8;4(2):206–13. doi: 10.1002/bco2.200 (PMC9931542; doi:10.1002/bco2.200)

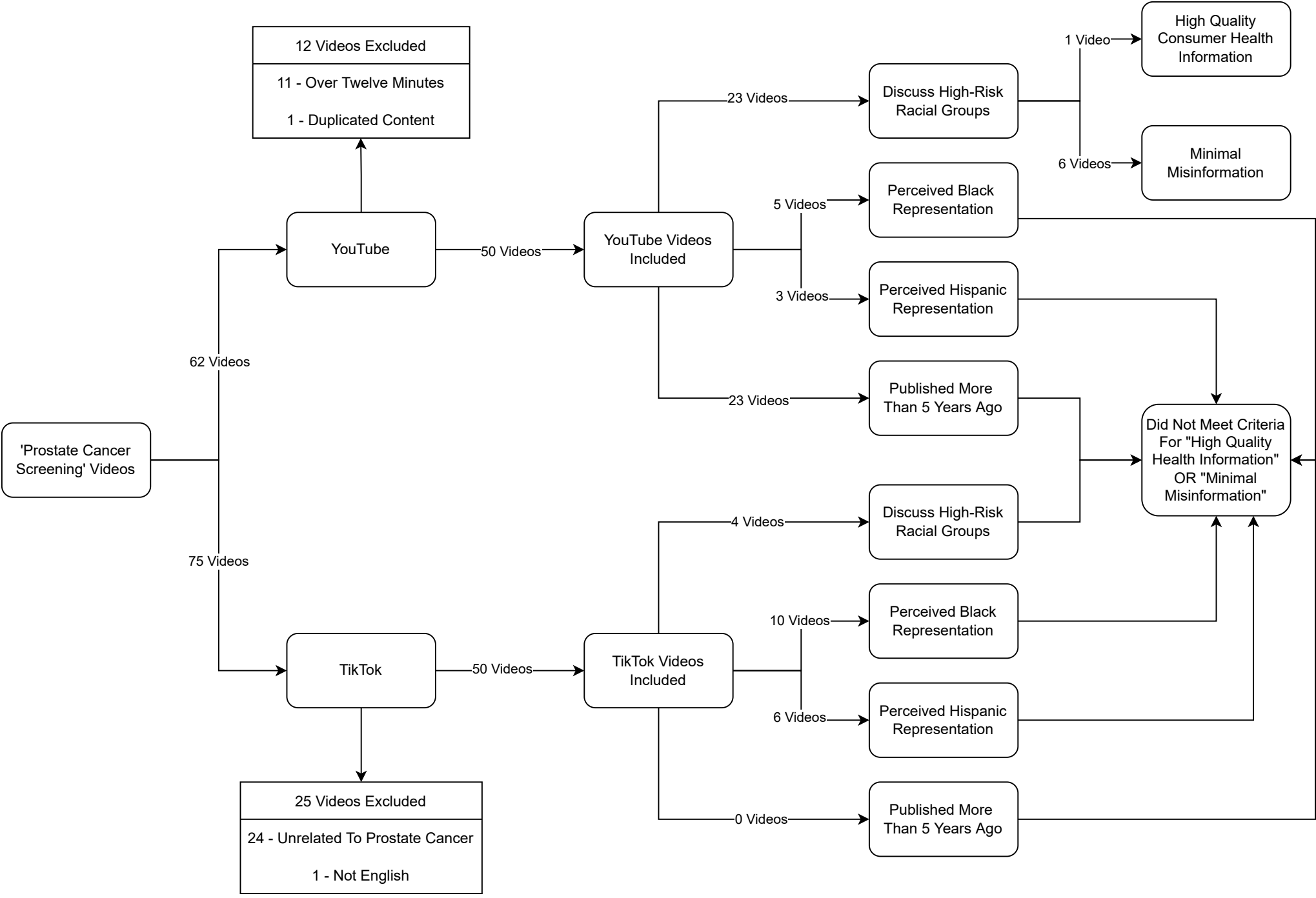

Supplement: Supplementary file 1 — Figure S1. Flowchart of video inclusion and breakdown of general findings on YouTube and TikTok [file BCO2-4-206-s001.pdf]
